# 4042 Enhancing Scientific Rigor, Reproducibility, and Reporting in Translational Science Training and Practice

**DOI:** 10.1017/cts.2020.387

**Published:** 2020-07-29

**Authors:** Roger Vaughan, Matthew Covey, Michelle Romanick, Anthony Carvalloza, Barry S. Coller

**Affiliations:** 1 Rockefeller University

## Abstract

OBJECTIVES/GOALS: Irreproducible and incompletely reported research lead to misallocated resources, wasted effort in pursing inappropriate avenues of investigation, and loss of public trust. To address this challenge, we employed a Team Science approach to create a multi-modal program to support Rigor, Reproducibility, and Reporting in Translational Science. METHODS/STUDY POPULATION: We conducted literature searches to reveal sources of irreproducibility and recommended corrective actions, invited leaders in the field to give lectures on opportunities to support reproducible science, and worked with the Rockefeller team science leadership group to instill an overarching rigor approach, infused into all training efforts. This multifaceted program was labeled R3 (R-cubed) for Enhancing Scientific Rigor, Reproducibility, and Reporting. RESULTS/ANTICIPATED RESULTS: Didactic Courses:

- Introduction to Biostatistics and Critical Thinking – focus on pitfalls in inferential statistics, consequences of poor research, and errors in published research.
- Scientific Writing – teaches methods and procedures in writing to ensure reproducibility. Lecture Series
- Established nine lectures on topics related to R3, including Data Management, Statistical Methods, Genomic Analyses, Data Repositories, Data Sharing, Pharmacy Formulation, and e-lab notebooks. Website
- Creating a comprehensive website as repository for research, methods, programs, updates, and improvements related to R3. KL2 Clinical Scholars Seminars and Navigation
- Scholars participate in seminars and tutorials to discuss opportunities to improve R3 across the research life-course.

DISCUSSION/SIGNIFICANCE OF IMPACT: Striving for research reproducibility takes focused energy, discipline, and vigilance, but the effort is worthwhile as rigorous and reproducible science is the prerequisite for successful translation of great discoveries into improved health. CONFLICT OF INTEREST DESCRIPTION: none

